# Role of integrins in the development of fibrosis in the trabecular meshwork

**DOI:** 10.3389/fopht.2023.1274797

**Published:** 2023-10-24

**Authors:** Jennifer A. Faralli, Mark S. Filla, Donna M. Peters

**Affiliations:** ^1^ Department of Pathology & Laboratory Medicine, University of Wisconsin School of Medicine and Public Health, Madison, WI, United States; ^2^ Department of Ophthalmology & Visual Sciences, University of Wisconsin School of Medicine and Public Health, Madison, WI, United States

**Keywords:** integrins, trabecular meshwork, fibronectin, extracellular matrix, fibrosis, TGFβ2, myofibroblast

## Abstract

Primary open angle glaucoma (POAG) is a progressive and chronic disease exhibiting many of the features of fibrosis. The extracellular matrix (ECM) in the trabecular meshwork (TM) undergoes extensive remodeling and enhanced rigidity, resembling fibrotic changes. In addition, there are changes associated with myofibroblast activation and cell contractility that further drives tissue fibrosis and stiffening. This review discusses what is known about the integrins in the TM and their involvement in fibrotic processes.

## Introduction

**Figure 1:**
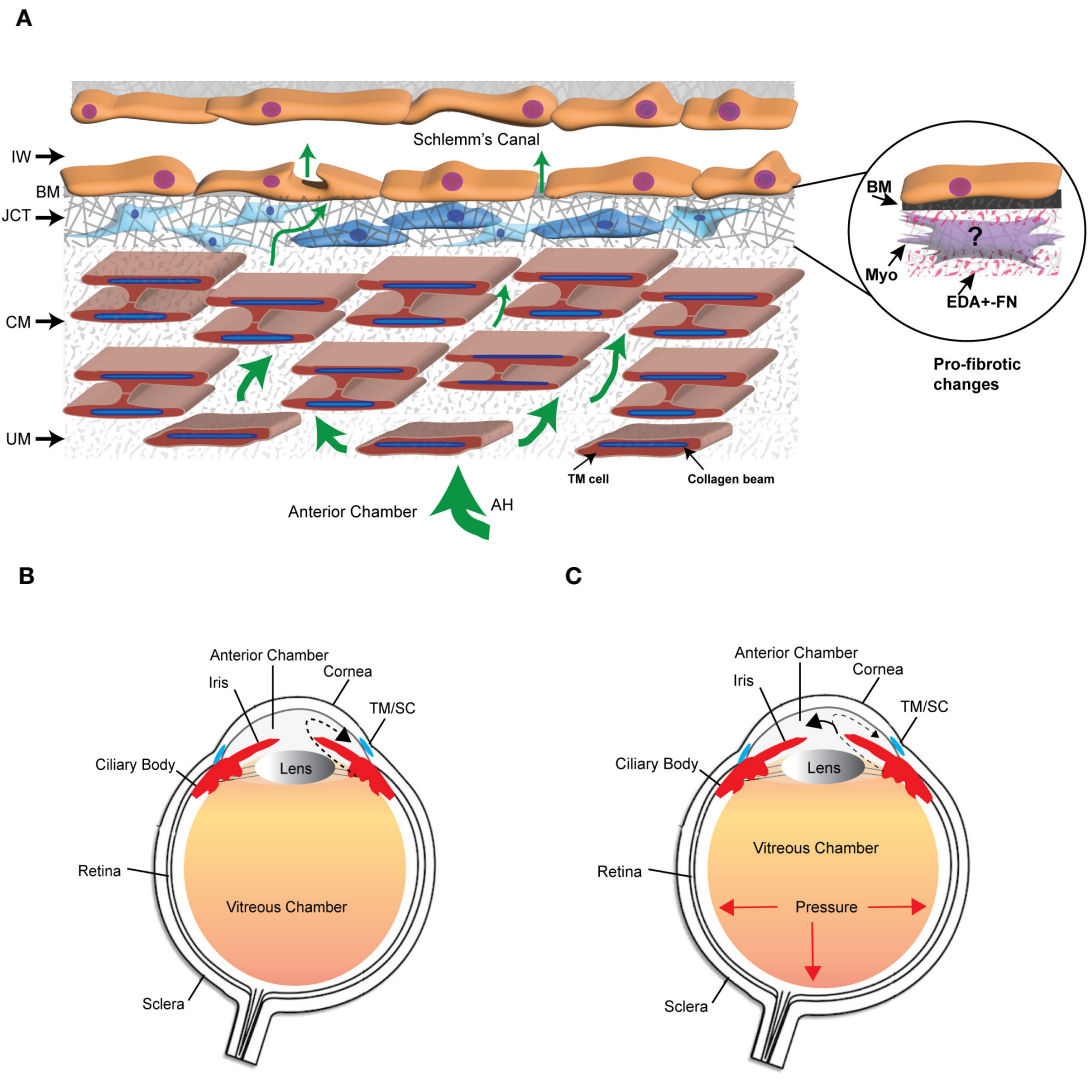
The trabecular meshwork/Schlemm’s canal (TM/SC) outflow pathway. **(A)** Aqueous humor (AH, green arrows) exits the anterior chamber through the uveoscleral meshwork (UM), corneoscleral meshwork (CM) and the juxtacanalicular tissue (JCT). It then crosses the basement membrane (BM) underlying the inner wall (IW) of Schlemm’s Canal to exit either paracellularly or transcellularly into the lumen of SC. The light and dark blue cells in the JCT indicate that the JCT consists of cells showing both fibroblastic and smooth muscle-like properties, respectively. The beams are connected to each other by cytoplasmic extensions between the TM cells surrounding the beams. The insert in the circle shows the profibrotic changes associated with POAG that may include the transition of TM cells in the JCT into myofibroblasts, the expression of the EDA+ isoform of fibronectin (FN), and the increased production of proteins in the BM of the IW wall. **(B)** Diagram of the whole eye showing the normal movement of aqueous humor (dashed arrow) from the ciliary body past the lens and iris into the anterior chamber and out through the TM/SC. **(C)** Diagram of the whole eye showing that profibrotic changes in the TM/SC shown in **(A)** would lead to a restriction in the movement of aqueous humor through the TM/SC (smaller arrow head) and an accumulation of more aqueous humor in the anterior chamber (larger arrowhead). This would result in increased pressure throughout the eye including in the vitreous chamber.

The extracellular matrix (ECM) is a dynamic network composed of structural and nonstructural proteins that are assembled into a tissue-specific architectural 3D scaffold that provides not only structural support for tissues but directs cell motility, survival, proliferation, and even cell death. In the trabecular meshwork (TM) in the anterior segment of the human eye, the ECM is primarily composed of different collagens (types I, III, IV, V, and VI), the glycoproteins fibronectin and laminin, and the proteoglycan hyaluronan. It also contains elastic fibers composed of fibrillin and elastin as well as multiple matricellular proteins whose expression may be transient. These ECM proteins can be found distributed throughout the various layers of the TM (1) (**Figure 1**). The uveal and corneoscleral meshworks form the trabecular lamellae and consist of collagen beams surrounded by a monolayer of endothelial-like TM cells on top of a basement membrane. The juxtacanalicular tissue (JCT) consists of cells exhibiting both fibroblastic and smooth muscle-like qualities (2, 3) loosely embedded in an ECM composed of different collagens, elastin fibers, fibronectin, hyaluronan, and various proteoglycans. Directly adjacent to the JCT is a monolayer of endothelial-like cells on top of a basement membrane that forms the inner wall of Schlemm’s Canal (SC). These last two layers of the TM are considered to be the major sites involved in regulating the outflow of aqueous humor and intraocular pressure (IOP) (4) and are also the regions where profibrotic changes are thought to lead to the pathogenesis of primary open angle glaucoma (POAG) (5).

Remodeling of the ECM in the TM is considered to be important in maintaining normal homeostasis of aqueous humor outflow through the TM (6, 7) and IOP. However, excessive and prolonged remodeling of the ECM in the JCT and inner wall of Schlemm’s canal (**Figure 1**) leads to a restriction in the outflow of aqueous humor that results in a buildup of fluid in the anterior chamber and an elevation in IOP. This is believed to trigger a pro-fibrotic like state resembling fibrosis that leads to the pathogenesis of POAG (8, 9). Transition into this pro-fibrotic state can start with either an age-related remodeling of the ECM that causes a general stiffening of the TM, thickening of the beams, loss of beam cells (5) or elevated levels of TGFβ2 in aqueous humor (10, 11). These changes which alter the mechanical properties of the TM (5, 12) activate signaling cascades that could cause the transdifferentiation of quiescent TM cells into myofibroblast-like cells. This process, termed endothelial-to-mesenchymal transition (EndoMT), can be driven by a variety of autocrine and paracrine signaling molecules including TGFβ, Wnt/β-catenin, Notch and/or inflammatory cytokines (13). EndoMT is also driven by the expression and/or structural stiffness of an isoform of fibronectin called EDA+ in the ECM (14). Myofibroblasts display a greater capacity to produce ECM proteins and contract (15, 16). The increased contractile properties of myofibroblasts further enhances the deposition of the ECM, notedly collagen and fibronectin thereby creating a feedback loop that further increases the rigidity of the ECM and pro-fibrotic activity of the tissue. These changes in the mechanical properties of a tissue are due in part to the activity of a family of transmembrane receptors called integrins (17, 18).

## Integrins in the TM

**Figure 2:**
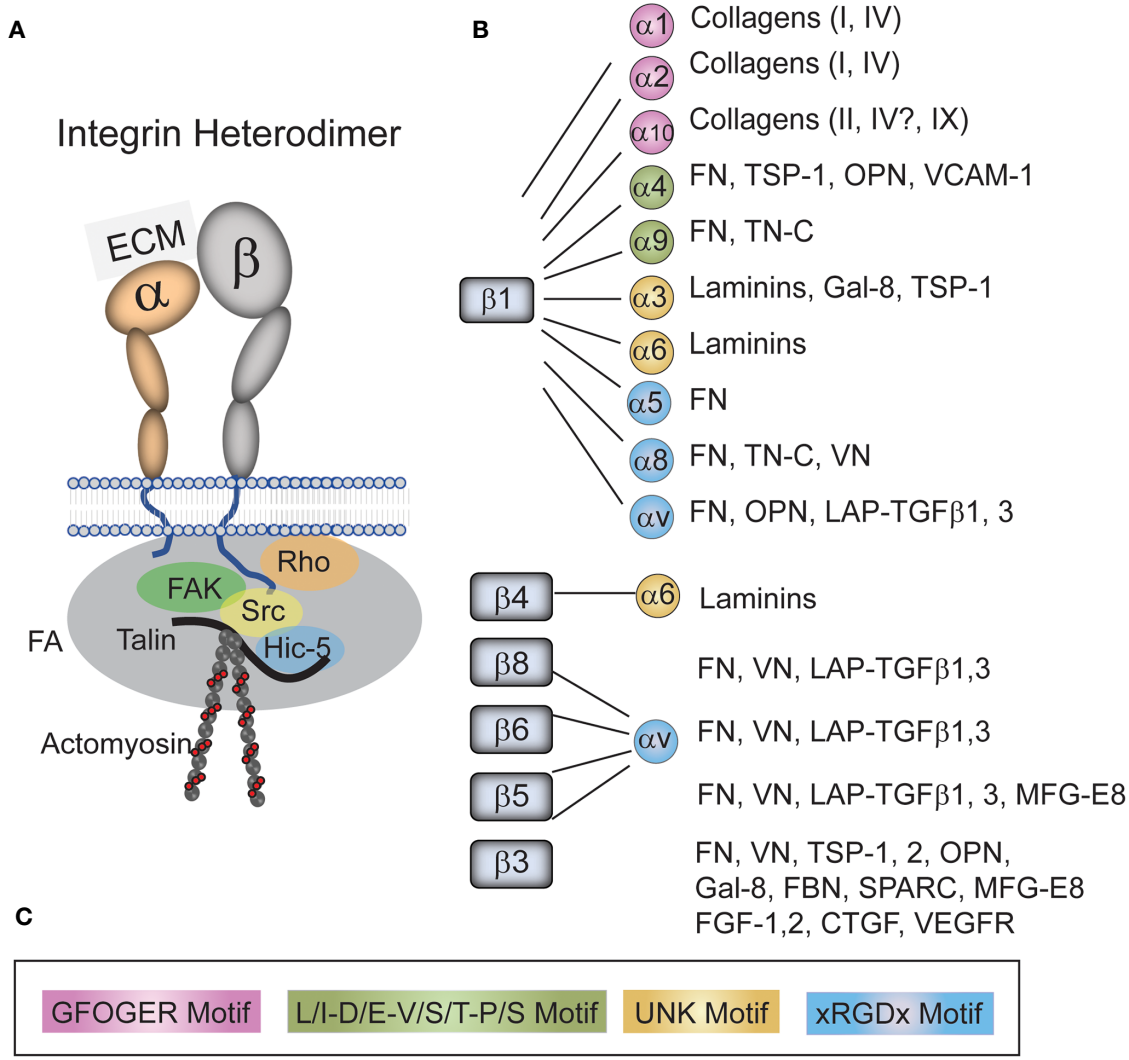
Integrins and their ligands found in the TM. **(A)** An integrin consists of an α- and β-subunit. The heterodimer functions to form a physical link between the extracellular matrix (ECM) and the cytoskeleton that acts as a signal transducer. The extracellular domain of the heterodimers bind to a variety of ECM proteins and growth factors found the TM/SC while the cytoplasmic tails of the heterodimer bind a variety of kinases (i.e. FAK, Src), adapter proteins (i.e. Talin, Hic-5) and members of the Rho family in a complex called a focal adhesion (FA). This complex of cytoplasmic proteins helps form a link between the tails of integrins and the actin cytoskeleton. It also connects integrins to signaling pathways including the Rho GTPase and TGFβ signaling pathways. **(B)** Integrins and their ligands found in the TM/SC. As shown, all integrins with the exception of α5β1 integrins bind multiple ligands. **(C)** The color-coded sequences in the box are the motifs that are recognized by each specific integrin in the various ligands. Fibronectin (FN), Thrombospondin-1 (TSP-1), tenascin-C (TN-C), Osteopontin (OPN), Vascular cell adhesion molecule (VCAM), galectin-8 (GAL-8), Vitronectin (VN), Latency-associated protein (LAP), Transforming growth factor β (TGFβ), Milk fat globule-EGF factor 8 (MFG-E8), Secreted protein acidic and rich in cysteine (SPARC), Fibrillin (FBN), Fibroblast growth factor (FGF), Connective tissue growth factor (CTGF), and Vascular endothelial growth factor receptor (VEGFR).

Each integrin is a heterodimer composed of an α- and a β-subunit (19–21) (**Figure 2**). In humans, there are 18 α and 8 β subunits which mix and match to form 24 unique receptors that have tissue-specific biological properties and show specificity for different ECM ligands. For instance, α5β1 integrin only binds fibronectin whereas α4β1 integrin binds fibronectin and VCAM. Although all integrins have been shown to mediate cell attachment to the ECM, several integrins also have distinct biological functions. For example, α5β1 integrin is best known for regulating fibronectin fibrillogenesis (22, 23) and αvβ5 integrin regulates phagocytosis (24, 25). A more detailed discussion of integrin structure and function in the TM can be found in a recent review (21).

At least 20 different integrins have been identified in the cells associated with the TM in the outflow pathway by either RNA or protein analysis. These integrins show a broad distribution and are found along the trabecular beams, in the JCT and in SC cells along the inner wall demonstrating that multiple integrins are expressed on cells throughout the TM/SC (25–27). The major integrins found in the JCT of the TM/SC by scRNA analysis of human tissues (3) appear to be αvβ5 and α9β1, whereas SC cells contain predominantly αvβ3, αvβ1, α5β1, α9β1, and α10β1. A complete listing of all the integrins found in this study can be found at the Broad Institute of MIT and Harvard Single Cell Portal^1^.

Although integrins are best known for mediating cell attachment to the ECM, integrins are also key partners in a number of signaling pathways involved in fibrosis including TGFβ signaling (28), formation of a fibronectin matrix (22, 29), myofibroblast formation (30), and activation of Rho GTPases (31). Integrins participate in these pathways via either direct interaction with receptors or through an association with intracellular cytoskeleton elements assembled into a signaling complex called focal adhesions (FAs). This latter association occurs through a variety of cytoskeletal linker proteins and kinases (i.e., FAK, Src, talin, paxillin, vinculin, etc.) which form a physical linkage that directly connects intracellular and extracellular structures (**Figure 2**). This puts integrins in a unique situation in that they exhibit bidirectional signaling. Integrins can convert extracellular biochemical signals generated from the proteins in the ECM into intracellular biochemical signals. They can also convert extracellular mechanical forces derived from the pulsatile motions of the TM (32) into intracellular biochemical signals. Both sets of intracellular biochemical signals can drive the differentiation of cells into myofibroblasts (30) and promote excessive ECM deposition during fibrogenesis. Alternatively, integrins can transmit intracellular, myosin-generated contractile forces to the outside of the cell that alter the architecture and signaling properties of proteins in the ECM (17, 33). These intracellular signals may include, but are not limited to, tyrosine phosphorylation of proteins such as paxillin and p130CAS (34–36), activation of protein tyrosine kinases such as FAK and Src (37), and activation of serine/threonine kinases such as Erk or Akt (38, 39). Extracellular signals, on the other hand, could include changes in the conformation of fibronectin (40) needed for fibrillogenesis (41).

## Integrins and Rho GTPases in the TM

Among the important signaling pathways controlled by integrins that are involved in fibrosis is the Rho GTPase pathway (31, 42). Rho GTPase pathways involving RhoA, Rac, and Cdc42 are essential in regulating the contractile (43, 44) and phagocytic (25) properties of the human TM. They are also needed for the enhanced contractile properties of myofibroblasts and the deposition of proteins associated with fibrosis into the ECM (45, 46). Integrins control GTPase mediated-processes by directing the localization and activation of Rho GTPases at the membrane (47). This is done by controlling the activity of guanine nucleotide exchange factors (GEFs) and GTPase activating proteins (GAPs) that control Rho GTPase activities (48). For instance, in human TM cells, activation of αvβ3 integrin uses the GEF Tiam1 to trigger the activity of Rac1 which promotes the reorganization of actin and smooth muscle α-actin (α-SMA) into crosslinked actin networks (CLANs) (49, 50). This network is frequently observed in glaucomatous cells and tissues and is believed to alter TM contractility (51, 52) and hence the mechanical properties of the TM/SC. Activation of Rac1 by αvβ3 integrin also leads to an inhibition of Rho-mediated phagocytosis (25, 53). In this scenario, αvβ3 integrin uses the GEFs Tiam1 and RhoG/ILK/ELMO2 rather than Trio to trigger Rac1 activity. Once activated, Rac1 could inhibit RhoA activity by upregulating a 190 RhoGAP (54).

RhoA, another member of the Rho GTPase family, plays a prominent role in promoting the contractile properties of the actomyosin network in the TM that control IOP. In human TM cells, constitutively active RhoA causes a significant increase in the formation of actomyosin networks, and in rodent models of ocular hypertension it causes an increase in α-SMA expressing myofibroblast-like cells (55–59). Thus, studies have shown that inhibiting the Rho-associated protein kinase (ROCK), a downstream effector of RhoA activity, is an effective treatment for lowering IOP in POAG (58, 59).

RhoA also leads to an increase in the deposition of ECM proteins especially fibronectin in human TM cells (55). In these studies the activity of RhoA, as well as the TGFβ/SMAD pathways, was controlled by the αvβ3 integrin, together with the subsequent recruitment of Hic-5 to FAs (57). The activation of αvβ3 integrin or overexpression of Hic-5 induced the cytoskeleton changes attributed to RhoA activity while the knockdown of Hic-5 suppressed TGFβ2-induced fibrogenic activity (57). Interestingly these studies found that activation of RhoA and the TGFβ/SMAD pathways occurred in the absence of TGFβ2. This suggests that integrin-mediated signaling may play an essential role in the TGFβ2-mediated activation of RhoA during fibrosis. This is not totally unexpected since studies have shown that integrin engagement plays a critical role in growth factor signaling including TGFβ signaling (60–63) and the subsequent activation of RhoA.

## Role of α5β1 and αvβ3 integrins in fibronectin matrix formation

**Figure 3:**
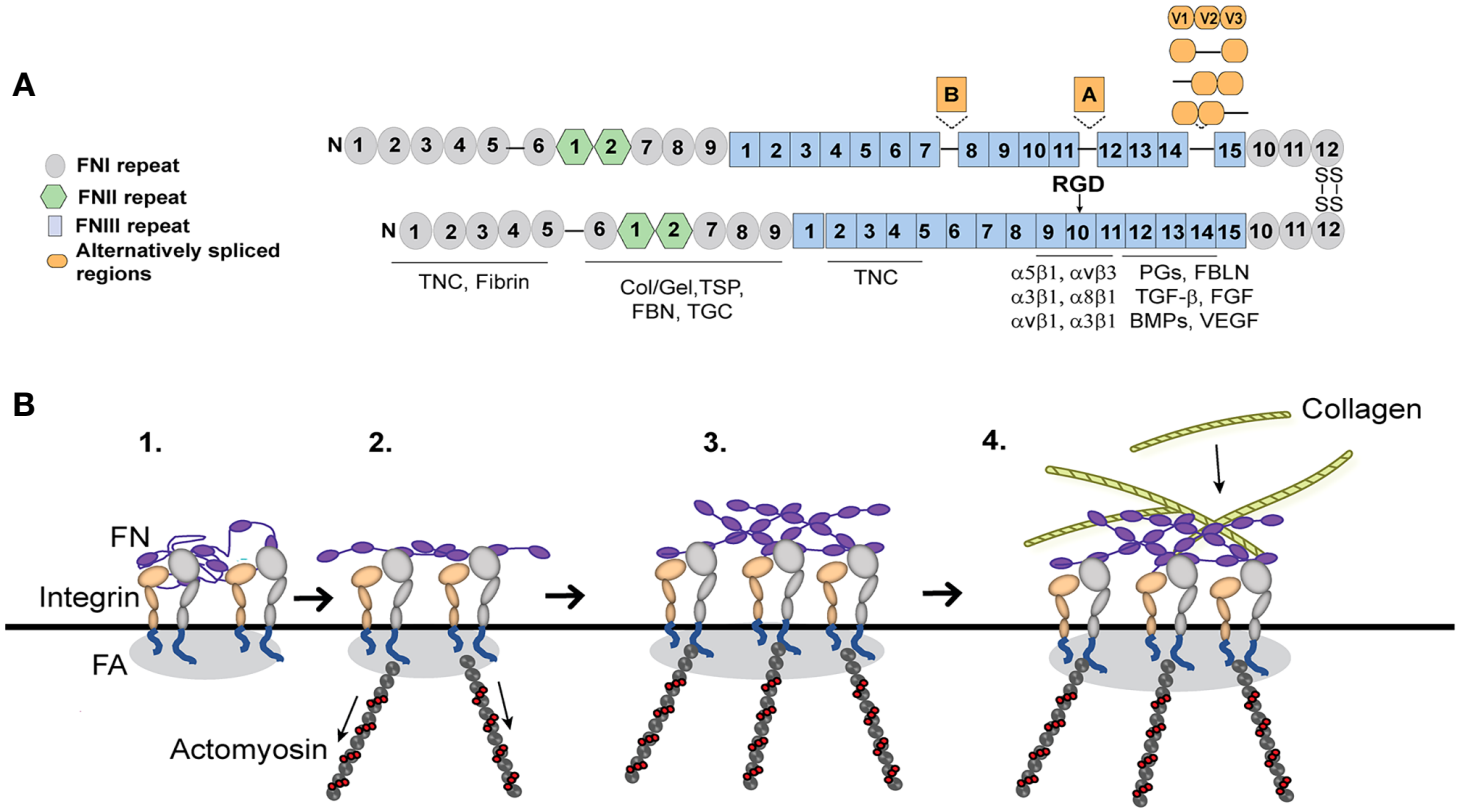
Fibronectin (FN) matrix assembly model. **(A)** Fibronectin consists of three repeating numbered modules (types I, II, III) and three alternatively spliced sequences. The ECM proteins that use fibronectin to be incorporated into the ECM are indicated below their fibronectin binding sites. The major integrin binding site (RGD) is in the 10^th^ FNIII repeat and binds the integrins indicated in the figure. Tenascin-C (TNC), Collagen/gelatin (Col/Gel), Thrombospondin (TSP), Fibrillin (FBN), Transglutaminase (TGC), Proteoglycans (PGs), Fibulin (FBLN), Transforming growth factor β (TGFβ), Fibroblast growth factor (FGF), Bone morphogenetic proteins (BMPs) and Vascular endothelial growth factor (VEGF). **(B)** Fibronectin, which is secreted as a globular dimer, binds an integrin in a focal adhesion (FA) on the cell surface (step 1). Contractile forces generated by the actomyosin cytoskeleton connected to the integrins (step 2) cause the fibronectin dimer to unfold. Fibronectin-fibronectin binding sites (purple ovals) mediate fibril formation (step 3). These fibronectin fibrils serve as a scaffold for the incorporation of other ECM components such as collagen fibrils (step 4).

Integrins play a critical role in fibrosis since they are responsible for the deposition and formation of fibronectin fibrils that direct and maintain the organization of the ECM. Fibronectin is a dimeric glycoprotein maintained by two disulfide bonds at its C-terminus that is composed of an array of repeating modular structures called repeats (**Figure 3A**). There are 12 type I repeats (FNI), 2 type II repeats (FNII), 15 type III repeats (FNIII) and a non-homologous variable (V) or type III connecting segment (IIICS) region. In addition, it can contain 2 additional alternatively spliced type III repeats referred to as EDA and EDB repeats. These repeating segments create functional domains that interact with multiple binding partners within the ECM that allow fibronectin to help mold and maintain the 3D-architecture of the ECM during fibrosis and incorporation of other proteins into the ECM (64–69).

Fibronectin fibrillogenesis plays a critical role in fibrosis and disruption of it attenuated fibrosis in multiple *in vivo* models of fibrosis including two different mouse models of kidney fibrosis (70, 71) and a model of liver fibrosis (72). It also prevented fibrosis during a mouse model of heart failure (73). In TM cells in culture, inhibition of fibronectin fibril formation also inhibited the incorporation of other matrix proteins such as type IV collagen, fibrillin and laminin into the ECM (74). Intriguingly, inhibition of fibronectin fibrillogenesis also appeared to promote the removal of existing fibronectin fibrils (74) and lowered IOP *in vivo* (75). This suggests that controlling this integrin mediated assembly of fibronectin fibrils may represent a way to control fibrosis in glaucoma.

The assembly of fibronectin into fibrils in TM cells is mediated by several fibronectin binding integrins (22, 41). The major integrin involved is the α5β1 integrin. α5β1 integrins promote fibril formation by binding the secreted soluble dimer and inducing a conformational change that exposes specific fibronectin–fibronectin binding sites (**Figure 3A**). These binding sites are needed for the assembly of fibronectin into an insoluble fibril that then acts as the scaffold for any subsequent matrix deposition (74). Among the sites involved in fibril formation are the amino terminus of fibronectin (76) and the heparin II binding domain (77) and blocking their binding activity has been shown to prevent fibril formation. Thus, inhibiting these interactions have proven an attractive mechanism to control fibril formation and fibrosis.

Integrins can control this process because they regulate the contractile forces of the actomyosin network that are used to unfold and stretch fibronectin so that fibronectin-fibronectin binding sites needed for fibril formation are exposed (**Figure 3**). In human TM cells as in other cell types, this process usually involves the GTPase RhoA (23, 40) which appears to be activated when the α5β1 integrin engages the fibronectin monomer. In TM cells activation of αvβ3 integrin, however, enhances the assembly of fibronectin into fibrils leading to an increase in the deposition of fibronectin fibrils. How activation of αvβ3 integrin enhances the α5β1 integrin-mediated process is unclear. Unlike the RhoA-mediated process involving α5β1 integrin, the αvβ3 integrin uses a RhoA/ROCK-independent process (23) since the process is unaffected by the ROCK inhibitor, Y27632. Thus, it is possible that activation of αvβ3 integrins may be activating pathways that are independent of RhoA/ROCK by either using the guanine nucleotide exchange factor GEF-H1/mDia (78) or the GTPase Rac1 (49, 79) to generate the contractile forces of the actomyosin network in the cells. Interestingly, GEF-H1 has been shown to regulate RhoA-dependent cell stiffening and rigidity (48) while the RhoA/ROCK pathway has been observed to control fibrotic activity in human TM cells in culture (56) and *in vivo* (57). This suggests that the two processes together may be increasing the contractile forces regulating fibril formation and targeting a specific GEF as well as ROCK could be an effective treatment for preventing any profibrotic changes during POAG (58, 59).

Intriguingly, fibronectin fibrils assembled by TM cells expressing constitutively activated αvβ3 integrin also contained higher levels of the alternatively spliced isoforms of fibronectin containing the EDA and EDB domains (23). These alternatively spliced domains are usually not expressed in adult tissue unless the ECM in the tissue is being remodeled. The inclusion of both the EDA+ and EDB+ alternatively spliced domains in fibronectin supports a more robust response to TGFβ signaling whereas fibrils containing only EDA+fibronectin promoted a weaker response to TGFβ (80). Inclusion of the EDA+ and EDB+ domains into fibronectin also affects the thickness, stiffness, and degree of branching of the fibril and the pore size of the fibronectin fibrillar network (80). The EDA domain is also involved in the transition of cells into myofibroblasts (14). These fibrils also exhibited an altered fibril conformation that resulted in the exposure of a buried domain known as the L8 epitope (**Figure 3**) which involves the GLN-690 in the first type III repeat (81). Hence changes in the expression or activity of integrins in TM cells are likely to profoundly affect the function and compliance of the ECM as well as modulate ECM-mediated signaling events (21, 80). This also suggests that during glucocorticoid-induced ocular hypertension or glaucoma, where αvβ3 integrins are likely to be overexpressed and active (82), the αvβ3 integrin may induce the formation of fibronectin fibrils that are more similar to a fibrotic-like ECM.

## Role of integrins in contractility and myofibroblast differentiation

In many tissue types, fibrosis involves the formation of myofibroblasts. Myofibroblast-like cells have been observed in the TM of young human eyes (83). These cells express α-SMA, a marker of myofibroblasts and are randomly distributed throughout the TM. Interestingly, their prevalence decreases with age, but their levels increase in eyes following treatments with corticosteroids. Myofibroblasts develop pronounced α-SMA-myosin bundles (stress fibers in cultured cells) that have increased contractile properties and can connect with the ECM via integrins at sites of large focal adhesions (16). Although the formation of these α-SMA positive stress fibers is a distinguishing feature of myofibroblasts that provides the contractile properties that play an important role in the fibrotic process, α-SMA is not essential for the development of a myofibroblast phenotype (16, 84).

**Figure 4:**
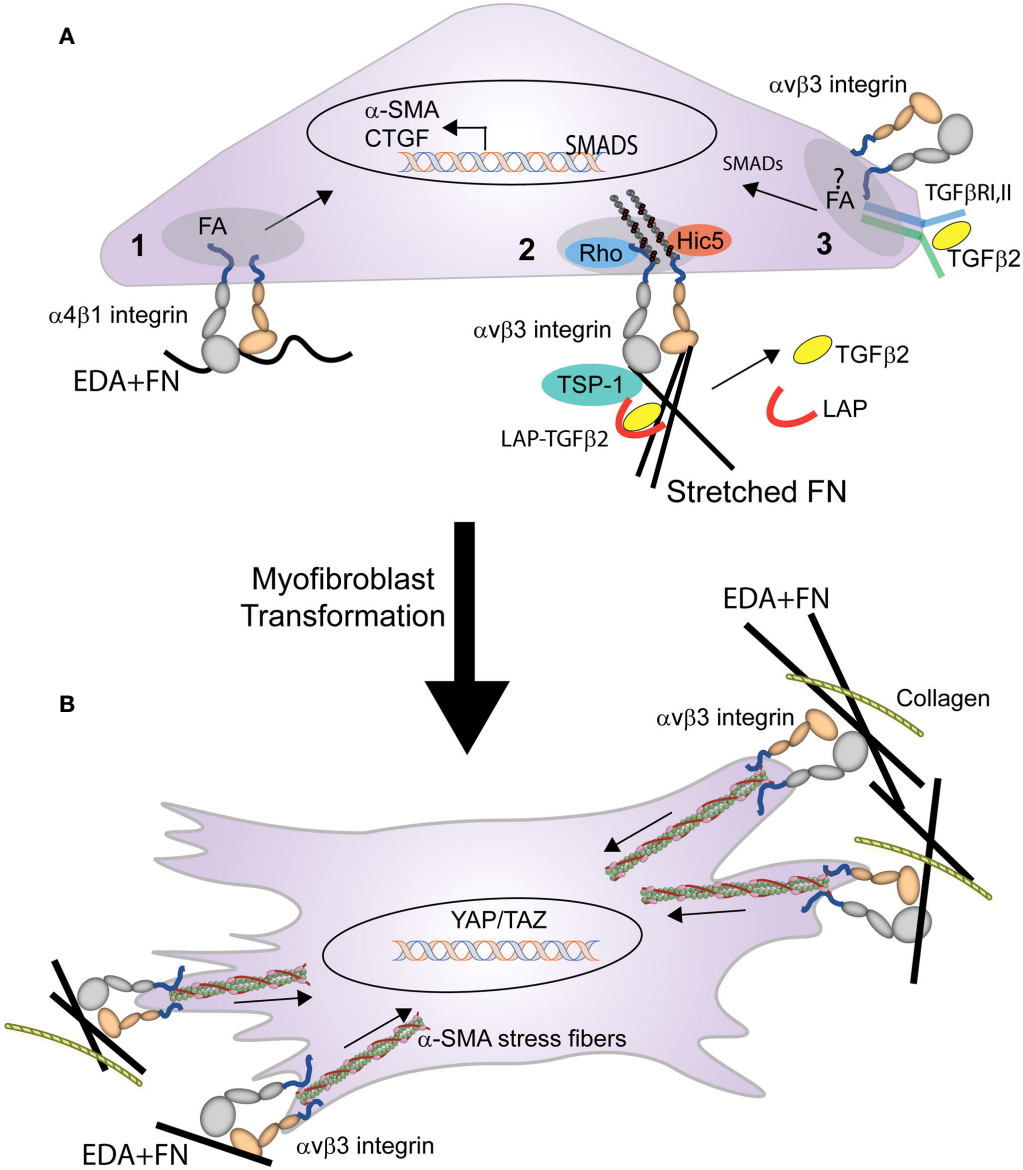
Role of integrins in the transformation of a cell into a myofibroblast. **(A)** Multiple integrin mediated processes trigger the early stages of myofibroblast formation. These processes include: (1) binding of EDA+ fibronectin to α4β1 integrin triggers expression of α-SMA, and (2) integrin mediated mechanical transduction activates RhoA, leading to assembly of actin stress fibers and contractile forces that promote the release of TGFβ from the LAP-TGFβ complex. (3) activated TGFβ1 (freed from LAP) binds to the TGFβRI/II complex stimulating SMAD intracellular signaling that promotes further expression of α-SMA, EDA+ fibronectin, and CTGF. This signaling, which triggers a feedback loop, may occur within the focal adhesion (FA) and be mediated by the specific integrin associated with TGFβRI/II complex in the FA. **(B)** Activation of the integrin-mediated processes in **(A)**, triggers the myofibroblast phenotype. The differentiated myofibroblast and its contractile properties are then sustained by the formation of supermature FAs containing αvβ3 integrin.

The transformation of a cell into a myofibroblast occurs in two stages (**Figure 4**). In the first stage, expression of the EDA+ isoform of fibronectin begins the transformation of cells into myofibroblasts by TGFβ1 (14). Recent studies in mice constitutively expressing EDA+ fibronectin support this idea and show that expression of EDA+ fibronectin enhances the TGFβ2-induced deposition of ECM in the TM causing an age-dependent elevation in IOP (85, 86). In fibroblasts this transformation appears to involve an interaction between the EDA domain in fibronectin and either the α4β1, α4β7 or α9β1 integrins (87, 88). Whether one of these integrins is also involved in the transformation of TM or SC cells into myofibroblasts remains to be determined.

Other integrins such as αv-containing integrins may help promote the transformation of a cell into a myofibroblast by triggering the release of TGFβ1 from the surrounding ECM (28, 89). Finally in human TM cells, interactions between αvβ3 integrin and connective tissue growth factor (CTGF) may play a role in the transformation of the TGFβ-induced myofibroblast phenotype observed in TM cells (90–92). CTGF has been shown to be a downstream mediator of TGFβ1 induced myofibroblast differentiation in NRK cells (93) and in TGFβ2-induced myofibroblast differentiation in TM cells (90). Interactions between CTGF and αvβ3 integrin may be involved in the TGFβ2-induced myofibroblast differentiation, since cyclic RGD peptides that bind αvβ3 integrin suppressed CTGF-induced fibrosis in animals and in TM cells in culture (92). This suggests that a CTGF/αvβ3 integrin mediated signaling pathway may participate in the transformation of a cell into a myofibroblast. In summary, combinatorial signaling pathways involving multiple integrins may be responsible for the profibrotic phenotype of the TM in POAG (94).

In the second stage of myofibroblast maturation, stress fibers containing α-SMA form and develop the contractile properties that can lead to further increases in the release of TGFβ2 from the ECM and enhanced ECM rigidity due to the dependence of integrins in the activation and release of TGFβ stored within the ECM. The formation and maintenance of these stress fibers as well the contractile force generated by the activation of αvβ3, α5β1, and αvβ5 integrins (84) within FAs (30) contributes to the continued transformation of the myofibroblast.

Maturation of FAs in myofibroblasts starts with the activation of a single integrin, αvβ3 integrin (95). Interactions between αvβ3 integrin (and possibly α5β1integrin) with their ECM ligand promote the development of super mature FAs that lead to the phosphorylation of the kinases FAK and Src and the subsequent activation of mechanosensitive signaling molecules such as MAPK, RhoA, and ROCK. It is the activation of these molecules that trigger α-SMA-containing stress fiber formation. These integrin-containing FAs also contribute to the transformation of the myofibroblast by serving as hubs for signaling pathways for the TGFβ1 receptor complex (16, 96). For instance, the TGFβ1 receptor complex appears to laterally associate in the fibroblast membrane with αvβ5 integrins which in turn promotes the activation of extracellular LAP-TGF-β1 and release of TGFβ stored in the ECM (28, 89, 97).

## αv-integrins mediate activation of TGFβ and signaling

Although TGβ1 is the most potent profibrotic cytokine known and the major driving force in the differentiation of myofibroblast phenotype (30), it does not appear to play a role in the pathogenesis of a fibrotic like state in POAG. Rather TGFβ2 appears to be predominantly involved in TGFβ induced POAG (11, 98) since numerous studies have now shown that sustained activation of ROCK by either TGFβ, CTGF or lysophosphatidic acid (LPA) in TM cells in culture or *in vivo* contributes significantly to creating the fibrogenic properties of the TM associated with POAG (11, 56, 57, 90, 91, 99–101). Not surprisingly, inhibition of ROCK signaling decreases ECM deposition in the TM and fibrosis but also increases aqueous humor outflow making it a therapeutic target for the treatment of POAG (46, 102).

In contrast to TGFβ1 where it is well established that the release of active TGFβ1 can be triggered by a variety of factors including αv-containing integrins and proteases (103–105), it is still unclear how TGFβ2 is activated. TGFβ2 is thought to be released by thrombospondin-1 (105) which is overexpressed in the TM from glaucomatous patients (106). Similar to the mechanisms governing the release of TGFβ1, release of TGFβ2 is also thought to involve a mechanical mechanism of TGFβ activation whereby integrin mediated contractile forces on the ECM could trigger the release of active TGFβ (103, 107), thereby possibly increasing TGFβ2 levels (**Figure 4**). This suggests a model whereby the combination of TGFβ, integrin-mediated contractility and changes in ECM expression could engage in a feed forward signaling loop (108) that leads to the pathogenesis of POAG. The enhanced ECM deposition by myofibroblasts leads to the production of a stiffer matrix with a higher mechanical load when compared with healthy ECM produced by fibroblasts (109). This mechanically stiffer ECM, in turn, leads to more efficient activation of TGFβ, and the increased levels of TGFβ further enhance myofibroblast differentiation leading to a positive feedback loop that sustains the profibrotic environment.

More recent studies in immortalized human TM cells in culture have suggested that the activation of the αvβ3 integrin leads to an increase in the expression of TGFβ2 mRNA and protein (110). Although, the mechanism behind this is still unclear, it appears that expression of TGFβ2 mRNA, like the β3 integrin subunit, may be a secondary glucocorticoid response modulated by calcineurin (CaN) and the transcription factor NFATc1 (82, 111). Activation of this CaN/NFATc1 pathway may be dependent on the TM cell cycle (111) since it was only activated when cells were in the proliferative state.

Finally, integrins could also play a role in TGFβ signaling by regulating the association of the two TGFβ receptors TGFβRI and TGFβRII into a complex (112) and their subsequent activation. In human lung fibroblasts, TGFBRI is enriched in FAs, while TGFBRII is selectively excluded. The oligomerization of the two receptors can be mediated by αvβ3 integrin which selectively recruits the TGFBRII receptor to interact with TGFβRI receptor in FAs (61). The αvβ3 integrin can also potentiate TGFβ signaling by controlling the activation state of TGFβRII. This is achieved through a direct interaction between αvβ3 integrin and the TGFβRII receptor that allows Src to phosphorylate and activate TGFβRII during the epithelial-mesenchymal transition of mammary epithelial cells (113). This interaction may explain the recent observation that a Src mediated TGFβ signaling pathway induced an elevation in IOP in a mouse model of ocular hypertension (114). Interestingly, recruitment of the α2β1 integrin to FAs could negatively regulate the tyrosine phosphorylation of TGFβRII through its recruitment of the phosphatase TCPTP into FAs (62). Thus, as with other growth factors, integrins have the capability of modulating TGFβ signaling (**Figure 4**).

## Conclusion

In summary, integrins play crucial roles in many important biological steps in fibrosis from the deposition of the ECM to the bioavailability of TGFβ and the contractile properties of myofibroblasts. Targeting integrins and the signaling pathways that they regulate could therefore be an important long-term antifibrotic strategy in chronic fibrotic diseases to preserve the function of the TM and restore homeostasis. Potential approaches to alleviate fibrosis in the TM would be to disrupt fibronectin fibril formation. This approach has proven successful *in vivo* using small fibronectin peptides to prevent fibrosis in vitreoretinopathy (115), but has not been pursued in the TM. Novel studies using recombinant integrin blocking antibodies (116) may also be another approach to reduce ECM production and/or the contractile properties of the tissue.

## Author contributions

JF: Writing – review & editing. MF: Writing – review & editing. DP: Conceptualization, Funding acquisition, Writing – original draft, Writing – review & editing.

## Footnotes

1
https://singlecell.broadinstitute.org/single_cell /study/SCP780
